# Impact of tea drinking upon tuberculosis: a neglected issue

**DOI:** 10.1186/s12889-015-1855-6

**Published:** 2015-05-29

**Authors:** Mengshi Chen, Jing Deng, Wufei Li, Dan Lin, Congxu Su, Mian Wang, Xun Li, Benjamin Kwaku Abuaku, Hongzhuan Tan, Shi Wu Wen

**Affiliations:** Department of Epidemiology and Health Statistics, School of Public Health, Central South University, Changsha, Hunan 410008 P. R. China; Hunan Children’s Hospital, Ziyuan RD 86, Changsha, Hunan 410007 P. R. China; Department of Nursing, Shaoyang Medical College, Shaoyang, Hunan 422000 P. R. China; Yueyanglou Center for Disease Control and Prevention, Yueyang, Hunan 414000 P. R. China; Department of Epidemiology, Noguchi Memorial Institute for Medical Research, College of Health Sciences, University of Ghana, P, O, Box LG581, Legon, Accra, Ghana; Department of Obstetrics & Gynecology and Department of Epidemiology & Community Medicine, University of Ottawa, The Ottawa Hospital 501 Smyth Road, Ottawa, Ontario Canada

**Keywords:** Tuberculosis, tea

## Abstract

**Background:**

Tuberculosis (TB) is a global public health issue posing serious harm to the human health. Many studies have suggested that smoking and excessive alcohol consumption are risk factors for TB. Laboratory evidence suggests that EGCG in tea leaves can arrest the growth of tubercle bacillus. Can drinking tea lead to decreased susceptibility of TB in humans?

**Methods:**

A total of 574 TB patients and 582 healthy controls were recruited to participate in this case–control study. Self-designed questionnaire was used to collect data. Unconditioned logistic regression analysis was conducted to identify the associations between tea drinking and TB.

**Results:**

Tea drinking has a negative association with TB, with OR = 0.583(0.423, 0.804) and *P* < 0.05. Drinking black tea, oolong and green tea are all negative association with TB, with OR being 0.683(0.517, 0.902), 0.674(0.508, 0.894) and 0.534(0.349, 0.817) respectively and *P* < 0.05. Trend *χ*^2^ test indicated a decreasing risk for TB with increased tea consumption, with *P* < 0.05.

**Conclusion:**

There is a significance negative association between tea drinking and TB. Promoting the consumption of tea as the daily drink among populations, particularly those with high TB risk, may reduce the incidence of TB in the populations.

## Background

Tuberculosis is a global public health issue posing serious harm to the human health. It is estimated that there were 8.6 million TB patients in 2012 globally [1]. China has the second highest TB burden in the world. According to the 5th TB epidemiological sampling survey in 2010 in China [2], the TB prevalence was 459/100,000 among people aged 15 years and above.

Many studies have suggested that smoking and excessive alcohol consumption are risk factors for TB. To date, there is no population study focused on the impact of tea drinking on Tuberculosis. The biological functions of catechins contained in tea leaves have drawn much interest recently. Tea is the second most highly consumed beverage after water [3]. Many studies have shown that tea drinking is beneficial for health in addition to preventing, and in some cases, alleviating certain health ailments, including inflammatory response [4–6], obesity [7–9], cardiovascular diseases [10–12], autoimmune diseases [13–17], neurodegenerative diseases [18] and tumors [19–22].

Laboratory evidence suggests that epigallocatechin-3-gallate (EGCG) in tea leaves can arrest the growth of tubercle bacillus by inhibiting the activity of InhA, the enoyl-acyl carrier protein reductase in tubercle bacillus [23]. It can also inhibit the survival of tubercle bacillus within macrophages by inhibiting the TACO (tryptophan-aspartate containing coat protein) gene transcription within macrophages [24]. The question then remains: Can drinking tea lead to decreased susceptibility of TB in humans? The case control study was used to explore the association between tea drinking and TB.

## Methods

### Sources of cases

Stratified sampling method was used to randomly select four county-level CDCs (i.e. Qidong County CDC, Yueyanglou District CDC, Yueyang County CDC and Hongjiang City CDC) using the random number table among 122 counties/cities/districts in Hunan Province, and then randomly select cases from all TB patients newly registered in 2009 by the four county-level CDCs. Cases of TB are diagnosed according to anatomical site of disease, bacteriological results, history of previous treatment and HIV status of the patient. Each of these key features of TB cases was discussed in World Health Organization Report [25]. Patients with HIV/TB co-infection were excluded. Patients who stopped receiving medications or were previously treated were also excluded.

### Sources of healthy controls

Stratified sampling method was used to randomly select one community health service center (i.e. Xingang Community Health Service Center) using the random number table among 14 community health service centers in Kaifu District, Changsha City, and then randomly select one community (i.e. Xin'ansi Community) from six communities covered by Xingang Community Health Service Center. The control group consists of healthy people without abnormalities in chest X-ray. Because the ratio of male to female TB patients was about 2.5:1 in Hunan [26], the healthy controls were selected from permanent residents in Xin'ansi Community by a gender-age frequency matching method. All controls were confirmed free from active TB. Although the cases and controls was selected from different district, both of them is Han nationality, all the districts with the same TB control policy and measures, very close distance (in Hunan province), and very similar economic level and life manners and customs in different district population, such as in tea drinking habit.

### Ethical statement

The work met relevant ethical guidelines. Written informed consent was obtained from all subjects of this research, in accordance with the guidelines of Central South University Ethics Review Committee. The protocol was also approved by Central South University Ethics Review Committee. Investigations were conformed to the principles outlined in the declaration of Helsinki.

### Data collection

Self-designed questionnaire was used to collect data. All the TB patients were asked to recall the situation of exposures when they came to CDCs for the confirmed diagnosis of TB. Data on exposure to tea drinking were collected through self-reporting by participants. In the survey, the respondents were asked if they are regular tea drinkers, if so the major concerns then centered around their tea drinking habits including frequency, duration and tea leaf choices. Questions about average monthly tea consumption include: how many grams of tea leaves a consumer buys every time, how many family members drink the tea, and how long it takes for them to finish it. Exposure to tea drinking is defined as at least one cup of tea drinking per week on average for over six months. Average monthly tea consumption is calculated by the duration of this consumption from a certain number of family members of a certain amount of tea leaves.

### Statistical analysis

Epidata3.0 was used to input data and SAS9.2 was used to analyze the data. Chi square test was conducted for the comparison of grouped data. Logistic regression was used for multivariate analysis. All tests of hypothesis were two tailed with a type 1 error rate fixed at 5 %.

## Results

The study participants included 574 TB patients and 582 healthy controls. The TB patient group and the healthy control group exhibited no difference in statistical significance (*P* > 0.05) in terms of sex, age, marital status, education background and alcohol drinking; while the difference in terms of BMI, smoking, history of BCG vaccination and cooking with solid fuel was statistically significant (*P* < 0.05); tea drinking has significant negative association with TB (OR = 0.650, P < 0.05) (Table 1).

**Table 1:** Univariate analysis of demographic characteristics and associated risk factors in TB

		TB Patients		Healthy controls		OR (95 % CI)
		*N* (%)		*N* (%)		
Sex	Male	427	74.39	410	70.45	
	Female	147	25.61	172	29.55	0.821(0.634,1.063)
Age (years)	19-30	73	12.72	85	14.60	Reference
	31-50	219	38.15	205	35.22	1.244(0.862,1.794)
	51-70	195	33.97	182	31.27	1.248(0.860,1.810)
	71-84	87	15.16	110	18.90	0.921(0.605,1.402)
Marital status	Married	403	70.21	397	68.21	
	Other	171	29.79	185	31.79	0.911(0.709,1.169)
Education background	Primary school or below	234	40.77	239	41.07	Reference
	Junior high school	178	31.01	182	31.27	0.999(0.759,1.314)
	Senior high school or above	162	28.22	151	25.95	1.096(0.823,1.458)
BMI	<18.5	208	36.24	199	34.19	Reference
	18.5-24.9	334	58.19	325	55.84	0.983(0.768,1.259)
	≥25	32	5.57	58	9.97	0.528(0.329,0.847)*
Smoking	No	242	42.16	312	53.61	
	Yes	332	57.84	270	46.39	1.585(1.257,2.000)*
Alcohol drinking	No	477	83.10	496	85.22	
	Yes	97	16.90	86	14.78	1.173(0.855,1.609)
History of BCG vaccination	No	462	80.49	416	71.48	
	Yes	112	19.51	166	28.52	0.608(0.462,0.799)*
cooking with solid fuel	No	234	40.77	312	53.61	
	Yes	340	59.23	270	46.39	1.679(1.330,2.119)*
Tea drinking	No	301	52.44	243	41.75	
	Yes	273	47.56	339	58.25	0.650(0.515,0.820)*

*P < 0.05

To exclude possible confounding and explore all the determinants meantime, multivariate unconditioned logistic regression analysis was conducted by using sex, age, marital status, education background, BMI, and alcohol drinking as the covariates, smoking, history of BCG vaccination, cooking with solid fuel, and tea drinking as independent variables. Results show that tea drinking and History of BCG vaccination had a negative association with TB (OR = 0.583 and 0.598), smoking and cooking with solid fuel was the risk factor of TB (OR = 1.589 and 1.490) (Table 2).

**Table 2:** Multivariate Analysis of Associated Risk Factors in TB

	Coefficient (*β*)	OR_ad_ (95 % CI)^#^
Tea drinking	−0.539	0.583 (0.423,0.804)*
Smoking	0.463	1.589 (1.179,2.142)*
History of BCG vaccination	−0.514	0.598 (0.428,0.836)*
cooking with solid fuel	0.399	1.490 (1.095,2.027)*

^#^ Multivariate logistic regression model was used to adjust the covariates of sex, age, marital status, education background, BMI, and alcohol drinking.

*P < 0.05

The study was also conducted to ascertain the associations of different classes of tea on Tuberculosis. Tea leaves can be classified as black tea (fermented tea leaves), oolong (semi-fermented tea leaves) and green tea (unfermented tea leaves). The results indicate that drinking black tea, oolong and green tea almost had same negative associations with TB, with OR being 0.683, 0.674, and 0.534, respectively (*P* < 0.05). (Table 3)

**Table 3:** The association between types of tea leaves and TB

Type of tea leaves	TB Patients		Healthy controls		OR_c_ (95 % CI)	OR_ad_ (95 % CI)^#^
	*N*	%	*N*	%		
Not drinking tea	301	52.44	243	41.75	Reference	Reference
Black tea	63	10.98	73	12.54	0.697 (0.478,1.016)	0.683 (0.517,0.902)*
Oolong	84	14.63	98	16.84	0.692 (0.494,0.969)*	0.674 (0.508,0.894)*
Green tea	126	21.95	168	28.87	0.605 (0.455,0.806)*	0.534 (0.349,0.817)*

^#^Multivariate logistic regression model was used to adjust the covariates of sex, age, marital status, education background, BMI, smoking, alcohol drinking, history of BCG vaccination and cooking with solid fuel.

*P < 0.05

Subjects who drank tea were categorized into three groups by monthly amount of tea drinking: 1-60 g group, 61-150 g group, and 151-300 g group, which would help investigate a dose–response relationship of tea consumption on TB. OR for 1-60 g group, 61-150 g group, 151-300 g group were 0.674, 0.619 and 0.564, respectively, with *P* < 0.05; trend *χ*^2^ test indicated a decreasing risk for TB with increased tea consumption, with *P* < 0.05, which showed significant dose–response relationship of tea consumption on TB (Table 4).

**Table 4:** The association between the quantity of tea consumption and TB

Tea consumption (g/month)	TB Patients		Healthy controls		OR_c_ (95 % CI)	OR_ad_ (95 % CI)^#^
	*N*	%	*N*	%		
Not drinking tea	301	52.44	243	41.75	Reference	Reference
1-60	94	16.38	106	18.21	0.716(0.517,0.991)*	0.674(0.472,0.962)*
61-150	98	17.07	124	21.31	0.638(0.466,0.874)*	0.619(0.478,0.801)*
151-300	81	14.11	109	18.73	0.600(0.430,0.837)*	0.564(0.415,0.766)*

Trend *χ* ^*2*^ = 12.784, P = 0.000

^#^Multivariate logistic regression model was used to adjust the covariates of sex, age, marital status, education background, BMI, smoking, alcohol drinking, history of BCG vaccination and cooking with solid fuel.

*P < 0.05

## Discussion

In this study, we found smoking, cooking with solid fuel had positive association with TB, but BCG vaccination and tea drinking had a negative association with TB. Many researches showed that smoking was an independent risk factor for tuberculosis (TB), and the risk of TB increases with increments of smoking, which support our result. Recent years, cooking with solid fuel has been regarded as another risk factor of TB, and has been confirmed in different populations [27–30]. The protection conferred by the BCG vaccine is significantly greater when the vaccine is administered to neonates or school children. In children, protection against pulmonary TB can reach up to 80 % [31], however, only 50 % of adults are protected [32]. Our result supported that BCG vaccine would reduce the risk of TB, which is consistency in univariate and multivariate analysis.

Our main finding is that tea drinking had a negative association with TB. Interestingly, green tea afforded the most evident protection against TB, compared to other types of tea. In addition, increasing tea consumption is associated with a decreased risk of tuberculosis, showed significant dose–response relationship.

Tea leaves have high concentration in tea polyphenols, primarily consisting of flavonoids, such as flavanol monomers and flavanol gallates. Flavanol monomers mainly contain catechin, epicatechin and epigallocatechin. Flavanol gallates mainly contain epicatechin gallate and epigallocatechin-3-gallate (EGCG). These substances are most prevalent in tea leaves, while EGCG exhibits the strongest biological activity. The content of catechins can account for 30-40 % of the dry weight of fresh tea leaves [6], while the content of EGCG accounts for 50-80 % of the total catechins in tea leaves [33]. Green tea is not fermented, and the content of the catechins contained in green tea is higher than that in semi-fermented (oolong) and fermented (black tea) tea leaves.

Numerous studies suggest that tea drinking helps to prevent obesity [7–9], cardiovascular diseases [10–12], autoimmune diseases [13–17], neurodegenerative diseases [18], tumors [19–22]. To our knowledge, this is the first study that discovered the negative association between tea drinking and TB in human population, moreover, showed significant dose–response relationship. The negative association of tea drinking and TB is postulated to occur via the following mechanisms: Firstly, the EGCG in tea leaves inhibit the growth of tubercle bacillus by inhibiting the activity of InhA, the enoyl-acyl carrier protein reductase [23, 34, 35]. Secondly, EGCG in tea leaves weakens the transcription of TACO genes in human macrophages by inhibiting the activity of SP1 transcription factors in TACO gene promoters, thereby weakening the expression of TACO genes, which in turn inhibit the survival of tubercle bacillus in macrophages [24]. Therefore, EGCG supplementation is of key significance for the prevention of tubercle bacillus infection. However, more researches in other population or more basic mechanism researches were required.

There are some limitations in our study. Firstly, we did not collected information on TB contact information. Secondly, the cases and controls in our study were sampled from different regions. Because the different sampling district have very similar background and condition; the possible impacts of non-genetic factors such as sex, age, marital status, educational background, BMI, smoking, alcohol drinking, history of BCG vaccination and cooking with solid fuel were adjusted by multivariate logistic regression; and dose–response relationship was observed. So the results observed in our study should be reliable.

## Conclusions

Tea drinking perhaps was a protective factor against Tuberculosis. Although substantial efforts are yet to be made to explore the mechanism of action, this study indicated that tea drinking perhaps could have very important effect on prevent and control TB infection. Tea is highly affordable, convenient and popular. Promoting the consumption of tea as the daily drink among populations, particularly those with high TB risk, may reduce the risk of TB in the population. Especially in developing countries with high TB prevalence, this approach may bring about significant social and economic benefits.
